# Controlled register‐based study of road traffic accidents in 12,651 Finnish cancer patients during 2013–2019

**DOI:** 10.1002/cam4.5444

**Published:** 2022-11-17

**Authors:** Marja‐Liisa Huuskonen, Tero Koistinen, Niina Sihvola, Inkeri Parkkari, Sanna Palovaara, Ville Kytö, Jussi Sipilä, Sirkku Jyrkkiö, Eetu Heervä

**Affiliations:** ^1^ Department of Traffic Medicine Turku University Hospital and University of Turku Turku Finland; ^2^ Finnish Motor Insurers' Centre Helsinki Finland; ^3^ Finnish Crash Data Institute Helsinki Finland; ^4^ Finnish Transport and Communications Agency Traficom Helsinki Finland; ^5^ Department of Oncology Turku University Hospital and University of Turku Turku Finland; ^6^ Heart Centre Turku University Hospital and University of Turku Turku Finland; ^7^ Department of Neurology Siun Sote, North Karelia Central Hospital Joensuu Finland; ^8^ Clinical Neurosciences Turku University Hospital and University of Turku Turku Finland

**Keywords:** cancer, glioma, motor vehicle accident, road traffic safety

## Abstract

**Background:**

Little controlled evidence exists on road traffic accident (RTA) risk among patients diagnosed with cancer, while clinicians are often requested to comment their ability to drive. The aim of this study was to evaluate RTA risk in a population‐based cohort of cancer patients living in Southwest Finland.

**Patients:**

All adult patients diagnosed with cancer in 2013–2019 were included. Acute appendectomy/cholecystectomy and actinic keratosis patients without cancer were selected from the same region as the control cohort. Participants were cross‐referenced to a national driving licence database, yielding 12,651 cancer and 6334 control patients with a valid licence. Due to marked differences in their clinical presentation, the cancer cohort was divided into nine cancers of interest (breast, prostate, colorectal, lung, melanoma, head & neck, primary brain tumours, gynaecological and haematological malignancies). The nationwide law‐regulated motor liability insurance registry was searched for all RTAs leading to injury with claims paid to not‐ or at‐fault participants. At‐fault drivers were verified based on sex and birth year.

**Results:**

During a median follow‐up of 34 months, 167 persons were at‐fault drivers in RTAs leading to injury. Among the nine cancers of interest, RTA risk did not differ from the control cohort. Among cancer patients, multivariable regression suggested male sex and opioid use, but not advanced cancer stage or given systemic therapy, as the most influential risk factors for RTA.

**Conclusions:**

Cancer diagnosis itself was not associated with increased RTA risk, but other associated symptoms, medications, comorbidities or specific cancer subgroups may.

## Graphical abstract

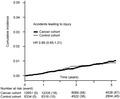

## 1 INTRODUCTION

Several guidelines for driving with cancer exists, mainly focusing on brain tumours (Supplementary material). However, poor awareness and adherence to these guidelines among cancer‐treating clinicians are reported.^1^, ^2^, ^3^ Cancer fatigue affects 50%–65% of cancer patients, especially during chemotherapy.^4^, ^5^ Fatigue, presenting as impairment of memory, processing speed, attention and executive functions, may be exacerbated by concomitant anxiety, insomnia, depression and sensation of pain.^4^, ^6^ This loss of cognitive function may be explained in part by major surgery, including general anaesthesia, following cancer diagnosis.^7^, ^8^ Regarding driving safety, cancer patients not only have an increased suicide risk especially among older men, but also in survivors of childhood cancer.^9^, ^10^, ^11^ Furthermore, opioid and benzodiazepine use impairs traffic safety, but this is generally attributed to older age and comorbidities. Regardless, cancer patients are often prescribed with opioids, and prescription opioids are associated with a roughly twofold increased risk of initiating a fatal car crash.^12^, ^13^, ^14^

Central nervous system malignancies cause seizures in 35%–70% of patients, who often present with resistance to antiepileptics.^15^ Moreover, seizures may occur in patients with many types of cancer, including in those without brain lesions.^16^

Studies specifically focusing on RTAs of cancer patients are scarce. A study from the Swedish cancer registry showed that cancer patients are at highest risk of injury for several weeks around their cancer diagnosis, but this was not the case for injuries sustained during transportation.^17^ Another study from the United States showed that comorbid conditions, especially heart disease and stroke but not cancer, increased RTA risk in elderly patients.^18^ Two small studies reported that patients with cancer fared worse in a virtual driving simulator,^19^, ^20^ but apart from these, no comparative traffic safety studies for cancer patients were found.

The aim of the current study was to use register‐based data to assess RTA risk in a cancer cohort and compare that to a non‐cancer cohort living in the same region.

## 2 MATERIALS AND METHODS

### 2.1 Ethics statement

This study was approved by the Institutional Review Board of Turku University Hospital (T190/2021) and the Finnish Transport and Communications Agency (Traficom, 309764/00.04.02.15/2021). Written consent is not required according to Finnish legislation on the secondary use of health data, when patients are not contacted.

### 2.2 Study cohorts

**FIGURE 1:**
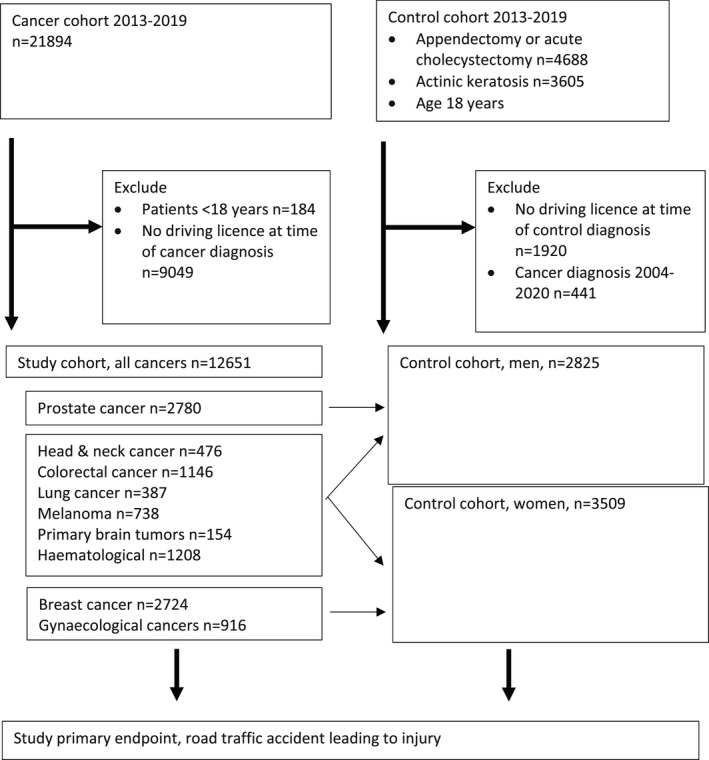
Flowchart of the study design.

Patients aged at least 18 years were selected from a previously described population‐based cohort of cancer patients treated at Turku University Hospital during 2013–2019.^21^ The control cohort consisted of patients treated in the same region at the same time for acute appendicitis or cholecystitis (Nordic operational [NOMESCO] codes JEA** and JKA**), or actinic keratosis (ICD‐10 code L57). Patients were cross‐referenced to national Traficom's driving licence registry, and those without a driving licence at the time of study entry were excluded (Figure 1).

Since demographics, prognosis and treatment modalities vary markedly between different types of cancer, we chose nine cancers of interest: head & neck (C00‐14,30–32), colorectal (C18–20), lung (C34), cutaneous melanoma (C43), breast (C50), gynaecological (C51–57), prostate (C61), primary brain tumour (C71) and haematological (C81–97). Advanced cancer was defined as presence of metastasis (C77–79) or stage III–IV haematological disease at diagnosis.

### 2.3 Traffic insurance data in Finland

Finland follows European Union directives, Finnish legislation, and Traficom guidelines for the approval and renewal of driving licences (supplementary material). Licences are approved by the police and since January 2013 have been electronically registered in Traficom's registry nationwide.

RTAs of study participants were identified from the insurance claim database of the Finnish Motor Insurers' Centre (FMIC). Motor liability insurance is mandatory for all vehicles registered in Finland and is provided by various insurance companies. The insurance claim database is nationally centralised to FMIC. Any person involved in the accident may report RTAs to the insurance company, which initially determines the person at fault after hearing from all participants and possibly the police. Responsibility for the RTA may also be shared if both sides violated traffic regulations, in which case the insurance claims are divided. Claims are paid from the insurance of the at‐fault person whenever an injury occurs or damage is caused to the not‐at‐fault person's property. Single vehicle crashes without injury are not compensated by the mandatory motor liability insurance.

Another person/company than the driver may hold the motor liability insurance itself. The person/company is nevertheless held accountable in the insurance claims, even if the RTA was caused by another person, unless there was criminal intent. Persons included in the current study were verified to be at‐fault by checking their sex and birth year against the FMIC motor liability insurance data.

### 2.4 Statistics

The primary endpoint is the first RTA leading to any injury, varying from mild to fatal. If a person was involved in multiple RTAs during the study period, the at‐fault RTA after and closest to the date of study entry was selected. In addition, vehicle‐damage‐only RTAs (without injuries) were collected as a secondary endpoint starting from 2017, when such data became electronically available. Only RTAs in which the study participant was the at‐fault driver, either fully or partially responsible, were included.

Follow‐up begins from the inclusion diagnosis (cancer/appendicitis/cholecystits/actinic keratosis) during which all patients were RTA free. All patients were followed up until death or the end of the study on December 2020. The nine cancers of interest were each compared with the control cohort—covering only women for breast and gynaecological cancers and men for prostate cancer. The RTA hazard ratios (HRs) were using Cox regression analysis with 95% confidence interval (CI) using SPSS version 26, and significant covariates were then entered into a multivariable model. Descriptive statistics were compared with Pearson's chi‐square (or Fisher's exact test when events were <5) or Tukey's analysis of variance.

## 3 RESULTS

### 3.1 Study population

Study participants with a valid driving licence are described in Table 1 and Table S1. At the time of study inclusion, cancer patients—apart from those with prostate cancer—had fewer driving licences (28%–74% without) compared to the controls (23%). Median follow‐up was 53 months for the control and 34 months for the cancer cohort.

**TABLE 1:** Description of study cohorts.

Group	Patients	Men: Women ratio	Median age (IQR), years	No. of comorbidities ≥3	Overall mortality, No. of deaths
Control (all)	6334	2825 (45%): 3509 (55%)	64 (51–76)	123 (2%)	109 (1%)
Control (women)	3509	—	59 (45–73)	43 (1%)	41 (1%)
Control (men)	2825	—	68 (58–78)	80 (3%)	68 (2%)
Breast	2724	19 (1%): 2705 (99%)	62 (54–70)*	8 (<1%)	35 (1%)
Prostate	2780	—	69 (64–74)*	76 (3%)	68 (2%)
Colorectal	1146	699 (61%): 447 (39%)*	69 (62–76)*	26 (2%)	90 (3%)*
Lung	387	244 (63%): 143 (37%)*	68 (62–74)*	13 (3%)	96 (24%)*
Head & neck	476	310 (65%): 166 (35%)*	65 (58–72)*	10 (2%)	25 (5%)*
Primary brain	154	90 (58%): 64 (42%)*	55 (41–69)*	9 (6%)*	28 (18%)*
Melanoma	738	391 (52%): 347 (48%)*	64 (54–74)	14 (2%)	24 (3%)*
Gynaecological	916	—	64 (56–72)*	8 (1%)	46 (5%)*
Haematological	1208	745 (62%): 463 (38%)*	63 (53–73)	31 (3%)	79 (7%)*

* Denotes statistical significance compared to control cohort, *p* < 0.01.

Abbreviation: IQR, interquartile range.

There were generally more women (55%) in the control cohort than the cohort with cancers of interest (35%–48%). Apart from melanoma and haematological malignancies, median age differences with the control cohort were observed, cancer patients being generally older. Diabetes and coronary disease were more common in cancer patients and sleep apnoea in controls. Epilepsy was common in brain tumour patients (40% vs. 1%) and cataract in colorectal, lung, and prostate cancer (14%–22% vs. 10%). Apart from breast and lung cancer, depression was more common in control patients. Strong opioids were more often prescribed for cancer patients, but wide variability was observed concerning mild opioids.

### 3.2 Road traffic accident rate

A total of 65 RTAs leading to injury (one fatal) were observed in the control group and 102 (four fatal) in the cancer group. RTA risk was not increased in any of the nine cancers of interest (Table 2). The cumulative incidence of RTAs during 1‐, 3‐ and 5‐year follow‐up was 0.1%, 0.7% and 1.9% in cancer patients and 0.2%, 0.8% and 1.6% in controls, respectively (Figure S1A). No difference in RTA risk was observed in the whole cancer group (HR 0.89 [0.65–1.21]) compared to controls.

**TABLE 2:** Road traffic accident (RTA) risk in the cancers of interest.

	Primary endpoint: RTA leading to injury HR (95%CI)	Persons followed (cancer/control)	Secondary endpoint: RTA without injury HR (95%CI)	Persons followed (cancer/control)
Breast cancer^a^	1.1 (0.6–2.1)	2724/3509	1.2 (0.6–2.3)	1262/1434
Prostate cancer^b^	1.0 (0.6–1.5)	2780/2825	0.9 (0.6–1.4)	1273/1218
Colorectal	1.3 (0.7–2.3)	1146/6334	1.3 (0.7–2.2)	572/2652
Lung	1.3 (0.4–4.0)	387/6334	0.6 (0.2–2.0)	278/2652
Head & neck	0.7 (0.2–2.2)	476/6334	1.6 (0.8–3.3)	236/2652
Primary brain tumours	No events in the cancer group	154/6334	No events in the cancer group	89/2652
Melanoma	0.3 (0.1–1.2)	738/6334	1.5 (0.8–2.8)	336/2652
Haematological	1.0 (0.5–1.9)	1208/6334	1.0 (0.6–1.9)	590/2652
Gynaecological^a^	0.9 (0.3–2.3)	916/3509	0.9 (0.3–2.3)	469/1434

^a^ Compared to women only.

^b^ Compared to men only.

Multivariable analysis (Table 3) showed that the dominant risk factor for RTA leading to injury in cancer patients was male sex and prescription of opioids; mild opioids increased and strong opioids decreased the observed RTA risk. Other univariable risk factors included age over 80 years, presence of retinopathy/cataract, or presence of three or more comorbidities. Cancer‐specific candidate risk factors, including the presence of advanced/metastatic disease, poor performance status, or systemic anticancer therapy initiated within 3 months of diagnosis did not increase RTA risk.

**TABLE 3:** Uni‐ and multivariable analysis of RTA risk leading to injury in cancer patients (*n* = 12,651). Significant (95%) results in bold.

	Univariable	Multivariable
Male sex	**2.10 (1.38–3.20)**	**1.94 (1.26–2.97)**
Category 1 versus 2 licence	1.34 (0.86–2.10)	
Age		
25 or younger	2.05 (0.50–8.47)	
>25 to 65	Reference	
>65 to 80	1.28 (0.84–1.95)	
older than 80	**2.06 (1.08–3.93)**	1.46 (0.77–2.75)
ECOG performance status 2+ versus 0–1	1.64 (0.74–3.60)	
Anticancer treatment given within 3 months of diagnosis^a^	0.82 (0.51–1.31)	
Advanced/metastatic cancer at diagnosis	1.36 (0.60–3.11)	
Comorbidities		
Diabetes	1.28 (0.73–2.26)	
Coronary disease	1.10 (0.57–2.10)	
Alcohol abuse	1.22 (0.39–3.86)	
Depression	0.99 (0.40–2.44)	
Epilepsy	1.54 (0.49–4.86)	
Dementia	0.66 (0.09–4.54)	
Cerebrovascular disease	1.21 (0.56–2.60)	
Retinopathy	**2.18 (1.16–4.07)**	1.62 (0.81–3.28)
Glaucoma	0.76 (0.19–3.09)	
Cataract	**1.72 (1.06–2.80)**	1.30 (0.74–2.29)
Sleep apnoea	1.25 (0.65–2.41)	
No. of comorbidities ≥3	**1.95 (1.04–3.64)**	1.28 (0.62–2.65)
Opioid use (mild)^b^	**2.26 (1.04–4.95)**	**2.31 (1.06–5.04)**
Opioid use (strong)^c^	**0.31 (0.14–0.71)**	**0.32 (0.14–0.74)**
Benzodiazepine use	0.84 (0.57–1.24)	
Smoking status		
Persistent versus never	0.73 (0.40–1.35)	
Former versus never	1.04 (0.63–1.71)	

^a^ Excluding hormonal therapy only.

^b^ Tramadol and codeine.

^c^ Morphine, buprenorphine, oxycodone, and fentanyl.

Cancer patients crashed less frequently during the night, but otherwise no differences in crash site, involved vehicle type, or crash timing were observed (Table S2).

The secondary study endpoint was RTAs without injuries (vehicle damage only) with median follow‐up of 29 months in the control and 23 months in the cancer cohort. During 2017–2020, a total of 148 RTAs without injuries occurred involving 6308 cancer patients and 59 RTAs involving 2652 control patients. No differences were observed in the rate of RTAs without injuries among the nine cancers of interest (Table 2, Figure S1B) compared to controls.

## 4 DISCUSSION

### 4.1 Cancer diagnosis not associated with an increased car crash risk

Our observational study design reflects the observed RTA number, practices of driving licence suspension, and patient self‐awareness in a controlled Finnish cohort of 12,651 cancer patients. Here, we observe the RTA rate over a period of 7 years and report no difference in RTA risk between nine cancer groups and one control group. Compared to the earlier literature,^17^, ^18^, ^19^, ^20^, ^22^ our study is the largest study with a control group focusing on cancer patients. Around 200 annual RTA deaths are recorded in Finland, and fortunately, RTAs leading to injury were few, highlighting the need for large datasets and extensive follow‐up. Finland has centralised national registries for both driving licences and motor liability insurance claims, which enabled this population‐based chart review study. Because any person can report an RTA based on vehicle registration number (licence plate), we estimate that most RTAs are captured in the nationwide insurance registry. We were able to differentiate driver responsibility based on sex and birth year in the registry and could confirm that the study participant was most likely the at‐fault driver. We also observed that among cancer patients, advanced stage at diagnosis, or use of anticancer treatment did not lead to an increased risk of RTA.

### 4.2 Role of opioids and comorbidities

Observed RTA risk was paradoxically lower in patients prescribed strong opioids but increased in those prescribed mild opioids. The use of strong opioids is linked to more aggressive and late‐stage cancer,^23^ which in turn may result in more careful and less frequent driving. Our study contradicts the report from the United States where prescription opioids were associated with increased risk of fatal car crash, but that study was conducted in an unselected population, while ours is among cancer patients.^14^ On the other hand, milder opioids are generally prescribed more frequently,^24^ and people may not recognise the risk of cognitive impairment associated with these drugs.

Generally cancer patients more often had characteristics and comorbidities associated with increased RTA risk, including male sex, as compared to controls.^18^, ^25^, ^26^ Since the number of accidents was low in the current study, we observed increased RTA risk only in select comorbidities. More importantly, we observed among cancer patients that the accident risk increased when three or more comorbidities were present (Table 2). Drivers under 25 years of age are also under‐represented in the current study and firm conclusions cannot be made. The same holds true, also for those over 80 years, which appeared to be the most risky age group among cancer patients.

### 4.3 Patients with brain tumours

The observation that none of the brain tumour patients crashed is probably because 66% of such patients were excluded from the study for not having a driving licence. According to Traficom instructions, the first seizure automatically results in a 1‐year suspension of the driving licence.

### 4.4 Limitations

We acknowledge that observational studies cannot conclude that cancer diagnosis does not increase RTA risk, but the observed RTA rate remains similar to that of the control group. Caution is advised, since the nine cancers of interest groups became relatively small, and specific treatments, rare cancers, or subgroups may further significantly increase the RTA risk. It remains challenging to assess personally driven kilometres among the study participants, that is exposure,^27^ as this information is not available in any registry. Therefore, we cannot rule out the possibility that cancer patients drive less and more carefully and limit their driving to essential trips only, as seen in patients with early Alzheimer's disease.^28^, ^29^, ^30^ Most cancer patients undergoing treatment in Finland are eligible for reimbursed taxi travel, which may further reduce the need to drive oneself.

We cannot either exclude that some control patients may be affected by even more severe medical conditions, than those listed in Table S1. Since driving conditions vary geographically and play an important role in road traffic safety,^31^ our results should be extrapolated with caution in different countries.

## 5 CONCLUSIONS

Acknowledging that cancer patients may drive less and more carefully due to awareness of their impaired health or following the advice of healthcare practitioners, we found no alarming signs that cancer patients present a marked threat to traffic safety. Specific subgroups of cancer patients may nevertheless have an increased risk for car crash, not detected in our study population. Physicians treating or following up cancer usually work at specialised clinics, and are encouraged to continuously monitor the overall traffic safety risk while taking comorbidities and side effects from cancer treatment into account.

## AUTHOR CONTRIBUTIONS

**Marja‐Liisa Huuskonen:** Conceptualization (equal); writing – original draft (equal); writing – review and editing (equal). **Tero Koistinen:** Conceptualization (equal); data curation (equal); investigation (equal); writing – original draft (equal); writing – review and editing (equal). **Niina Sihvola:** Conceptualization (equal); data curation (equal); investigation (equal); writing – original draft (equal); writing – review and editing (equal). **Inkeri Parkkari:** Conceptualization (equal); data curation (equal); investigation (equal); writing – original draft (equal); writing – review and editing (equal). **Sanna Palovaara:** Conceptualization (equal); writing – original draft (equal); writing – review and editing (equal). **Ville Kytö:** Conceptualization (equal); writing – original draft (equal); writing – review and editing (equal). **Jussi Sipilä:** Conceptualization (equal); writing – original draft (equal); writing – review and editing (equal). **Sirkku Jyrkkiö:** Conceptualization (equal); project administration (equal); writing – original draft (equal); writing – review and editing (equal). **Eetu Heervä:** Conceptualization (equal); data curation (equal); formal analysis (lead); investigation (lead); project administration (lead); supervision (lead); visualization (lead); writing – original draft (equal); writing – review and editing (equal).

## FUNDING INFORMATION

This research did not receive any specific grant from funding agencies in the public, commercial, or not‐for‐profit sectors.

## CONFLICT OF INTEREST

Jussi Sipilä has received honoraria (Merck, Pfizer, Sanofi) and a consultancy fee (Rinnekoti Foundation), and holds shares in the Orion Corporation. No potential competing interest was reported by the other authors.

## Supporting information


Supplementary Table 1
Supplementary Table 2

Supplementary Figure 1

## Data Availability

The authors constructed the study database themselves by combining multiple raw data registries at Turku University Hospital. According to Finnish legislation of secondary use of health data and secure storage of data, all requests to share health data should be directed at Findata (https://findata.fi/en/), referring to the above‐mentioned permission codes. The data are not publicly available due to their containing information that could compromise the privacy of research subjects.
