# Supplementary material for: Controlled register‐based study of road traffic accidents in 12,651 Finnish cancer patients during 2013–2019
Source: Cancer Med. 2022 Nov 17;12(6):7406–13. doi: 10.1002/cam4.5444 (PMC10067070; doi:10.1002/cam4.5444)
Supplement: Supplementary file 1 — Supplementary Table 1 Supplementary Table 2 [file CAM4-12-7406-s002.docx]

**Huuskonen et al.**

**Controlled register-based study of road traffic accidents in 12651 Finnish cancer patients during 2013-2019**

**Supplementary Material**

**Online guidelines (accessed Oct 2022)**

Finland, TRAFICOM guidelines for driving licence: <https://www.traficom.fi/sites/default/files/media/regulation/FTD_Guidelines_Traficom_2021_EN.pdf>

Australian guidelines on brain tumors and driving:

<https://www.cancercouncil.com.au/brain-cancer/living-with-a-brain-tumour/driving/>

https://www.nsw.gov.au/driving-boating-and-transport/driver-and-rider-licences/health-conditions-and-disability/fitness-to-drive/medical-conditions-illness-and-injuries

United Kingdom and North Ireland

https://www.cancerresearchuk.org/about-cancer/brain-tumours/living-with/driving

<https://assets.publishing.service.gov.uk/government/uploads/system/uploads/attachment_data/file/1084397/assessing-fitness-to-drive-may-2022.pdf>

Spain, lung cancer and driving

<https://www.fundacionmapfre.org/en/education-outreach/road-safety/mobility-safe-health/clinical-topics-and-safe-driving/respiratory-disease-driving/lung-cancer/>

Ireland

<https://www.rsa.ie/services/licensed-drivers/medical-fitness>

United States

https://www.fmcsa.dot.gov/sites/fmcsa.dot.gov/files/docs/mission/advisory-committees/mrb/83401/fmcsamedicalexaminerhandbook_0.pdf

**Material and methods**

An EU category 1 driving licence (for vehicles with mass <3500kg) may be applied for after the 18^th^ birthday, and category 2 (mass >3500kg) at the age of 21 or 24 depending on the vehicle and professional training of the driver. All new driving licences issued after January 2013 are valid for a fixed period as follows: Passenger car, motorcycle, moped, and tractor driving licences: 15 years. After the 65th birthday, the driving licence will be valid for a maximum of 5 years at a time. Lorry and bus/coach driving licences are valid for a period of 5 years at a time. After the 68th birthday, the driving licence is valid for a maximum of 2 years at a time. Prior to 2013, driving licences were valid until the 70^th^ birthday, except for lorry/bus licences. Conditional driving licences, such as driving during daylight only, are not applicable in Finland.

A medical report no more than 6 months old must be supplied when renewing a driving licence by everyone who will be: 1) over 70 years old when they renew their current category 1 licence; 2) over 45 years old when their category 2 licence expires; and 3) renewing their driving licence after the expiry date.

A driving licence may be suspended by the police, either due to traffic regulation violations or a written report from any physician. Physicians are mandated by law to report a driving licence suspension to the police if the medical condition impairing the ability to drive is permanent and has been diagnosed for at least 2 years (changed to 6 months since 2016). In practice, a physician’s suggestion to suspend a driving licence is always implemented by the police. For shorter or non-permanent suspensions due to medical illness, the decision is given verbally and noted in the medical records in an un-structured manner, making a register-based approach impossible. The licence may be reinstituted by the police upon provision of a new medical report showing recovery from a previous condition. A driver may also voluntarily suspend their own driving licence.

The at-fault driver may appeal to the civil court or specific crash board concerning the insurance company’s decision of being at-fault at RTA.

**Prescription data**

Opioid use has been defined as prescription of ATC code N02A* since 2013, when prescription data became electronically available. Tramadol and codeine are classified as mild opioids and oxycodone, morphine, fentanyl, and buprenorphine as strong opioids. Benzodiazepines are defined as ATC codes N05B* and N05C*, excluding melatonin.

**Results**

Observed RTAs led to permanent suspension of the driving licence in 10 cancer and seven control patients.

**Supplementary Table 1.** In-depth baseline demographics.

| **Baseline demographics** | **Control group (all)** | **Breast cancer** | **Prostate cancer** | **Colorectal cancer** | **Lung cancer** | **Head & neck** | **Primary brain tumour** | **Cutaneous Melanoma** | **Gynaecological** | **Haematological** |
| --- | --- | --- | --- | --- | --- | --- | --- | --- | --- | --- |
| Patients | 6334 | 2724 | 2780 | 1146 | 387 | 476 | 154 | 738 | 916 | 1208 |
| **Age** |  |  |  |  |  |  |  |  |  |  |
| 25 or younger | 183 (3%) | <5 (<1%) | 0 | <5 (<1%) | <5 (<1%) | <5 (<1%) | 10 (6%) | 9 (1%) | <5 (<1%) | 51 (4%) |
| >25 to 65 | 3151 (50%) | 1682 (62%) | 777 (28%) | 423 (37%) | 130 (34%) | 241 (51%) | 99 (64%) | 390 (53%) | 491 (54%) | 622 (51%) |
| >65 to 80 | 2309 (36%) | 918 (34%) | 1729 (62%) | 570 (50%) | 219 (57%) | 198 (42%) | 42 (27%) | 280 (38%) | 372 (41%) | 464 (38%) |
| Older than 80 | 691 (11%) | 123 (5%) | 274 (10%) | 151 (13%) | 36 (9%) | 35 (7%) | <5 (<1%) | 59 (8%) | 49 (5%) | 71 (6%) |
| **Comorbidities** |  |  |  |  |  |  |  |  |  |  |
| Epilepsy (G40) | 88 (1%) | 26 (1%) | 34 (1%) | 15 (1%) | 17 (4%) | 11 (2%) | 62 (40%) | 15 (2%) | 0 (0%) | 15 (1%) |
| Diabetes (E10-14) | 529 (8%) | 169 (6%) | 342 (12%) | 168 (14%) | 54 (14%) | 71 (15%) | 11 (7%) | 64 (9%) | 68 (7%) | 144 (12%) |
| Coronary disease (I21-25) | 477 (8%) | 92 (3%) | 385 (14%) | 126 (11%) | 60 (16%) | 43 (9%) | 5 (3%) | 58 (8%) | 27 (3%) | 99 (8%) |
| Cerebrovascular disease (I60-63,I69) | 277 (4%) | 94 (4%) | 220 (8%) | 88 (7%) | 38 (10%) | 24 (5%) | 18 (12%) | 32 (4%) | 24 (3%) | 68 (6%) |
| Alcohol abuse (F10) | 175 (3%) | 30 (1%) | 83 (3%) | 30 (3%) | 25 (6%) | 21 (4%) | 5 (3%) | 10 (1%) | 7 (1%) | 20 (2%) |
| Dementia (F00-03,G30) | 36 (1%) | 30 (1%) | 49 (2%) | 28 (2%) | <5 (1%) | 6 (1%) | 0 | 11 (2%) | <5 (<1%) | 15 (1%) |
| Depression (F32-33) | 537 (8%) | 183 (7%) | 69 (2%) | 38 (3%) | 31 (8%) | 25 (5%) | 12 (8%) | 36 (5%) | 47 (5%) | 63 (5%) |
| Retinopathy (H34-36) | 276 (4%) | 108 (4%) | 173 (6%) | 69 (6%) | 24 (6%) | 17 (4%) | <5 (1%) | 36 (5%) | 31 (3%) | 57 (5%) |
| Glaucoma (H40) | 146 (2%) | 71 (3%) | 79 (3%) | 31 (3%) | 9 (2%) | 5 (1%) | <5 (2%) | 18 (2%) | 14 (2%) | 20 (2%) |
| Cataract (H25) | 680 (11%) | 246 (9%) | 372 (14%) | 198 (17%) | 86 (22%) | 45 (9%) | <5 (3%) | 74 (10%) | 65 (7%) | 119 (10%) |
| Sleep apnoea (G47.3) | 636 (10%) | 147 (5%) | 302 (11%) | 87 (8%) | 34 (9%) | 34 (7%) | 10 (6%) | 61 (8%) | 32 (3%) | 95 (8%) |
| **Opioid prescription** | **Control group (all)** | **Breast cancer** | **Prostate cancer** | **Colorectal cancer** | **Lung cancer** | **Head & neck** | **Primary brain tumour** | **Cutaneous Melanoma** | **Gynaecological** | **Haematological** |
| Strong* | 549 (9%) | 219 (8%) | 221 (8%) | 392 (34%) | 156 (40%) | 218 (46%) | 15 (10%) | 59 (8%) | 272 (30%) | 249 (20%) |
| Mild ** | 1126 (18%) | 406 (15%) | 818 (29%) | 112 (10%) | 55 (14%) | 110 (23%) | 25 (16%) | 122 (17%) | 91 (10%) | 223 (18%) |
| Benzodiazepines | 2558 (40%) | 1263 (46%) | 988 (36%) | 679 (59%) | 337 (87%) | 293 (62%) | 120 (78%) | 239 (32%) | 587 (64%) | 768 (64%) |
| **Driving licence** | 8254 | 3782 | 3491 | 2078 | 1514 | 888 | 452 | 1027 | 1640 | 2039 |
| Category 1 | 5251 (63%) | 2559 | 1942 | 931 | 300 | 365 | 122 | 586 | 866 | 907 |
| Category 2 | 1083 (13%) | 165 | 838 | 215 | 87 | 111 | 32 | 152 | 50 | 301 |
| No licence | 1920 (23%) | 1058 (28%) | 711 (20%) | 932 (45%) | 1127 (74%) | 412 (46%) | 298 (66%) | 289 (28%) | 724 (44%) | 831 (41%) |

*morphine, buprenorphine, oxycodone, fentanyl **codeine, tramadol

**Supplementary Table 2:** Details of all RTAs leading to injury. For descriptive purposes, the table includes also RTAs occurring in persons driving without a valid licence during the study period, which is illegal, but happens nevertheless. Data is self-reported by persons involved in the accident, including police, and includes crashes among patients without a valid driving licence at the time of study entry. * denotes statistical significance chi-square p < 0.05.

|  | Control group RTAs (n=72) | Cancer group RTAs (n=119) |
| --- | --- | --- |
| Winter (November-March) | 25 (35%) | 50 (42%) |
| Monday-Friday | 57 (80%) | 98 (82%) |
| Alcohol detected | 1 (2%) | 1 (1%) |
| RTA led to police investigation | 7 (10%) | 24 (20%) |
| Driver fully responsible for the RTA | 50 (68%) | 78 (64%) |
| Multiple persons injured | 15 (19%) | 29 (24%) |
| Insurance claim median (IQR), euro | 498 (166-3196) | 712 (177-4984) |
| Excluded from the study, i.e., no driving licence at study entry | 7 (10%) | 17 (14%) |
| **RTA site** |  |  |
| Intersection | 23 (32%) | 25 (21%) |
| Straight road | 23 (32%) | 61 (52%) |
| Turning road | 6 (8%) | 14 (12%) |
| Parking area | 12 (17%) | 9 (8%) |
| Other | 7 (10%) | 8 (7%) |
| **Vehicle driven** |  |  |
| Passenger car | 57 (80%) | 97 (83%) |
| Truck/lorry | 2 (3%) | 3 (3%) |
| Moped or motorcycle | 9 (13%) | 14 (12%) |
| Other | 3 (4%) | 3 (3%) |
| **Victim involved** |  |  |
| Passenger car | 18 (26%) | 41 (35%) |
| Truck/lorry | 2 (3%) | 3 (3%) |
| Moped or motorcycle | 2 (3%) | 3 (3%) |
| Pedestrian | 1 (2%) | 3 (3%) |
| Bicycle | 9 (13%) | 9 (8%) |
| Single-vehicle RTA | 35 (50%) | 53 (45%) |
| Other | 3 (4%) | 5 (4%) |
| **RTA details (with missing data)** |  |  |
| Night time (hours 22-07)* | 8 (11%)* | 4 (3%)* |
| Day time (hours 08-21) | 49 | 94 |
| Urban area | 44 (61%) | 67 (56%) |
| Rural area | 27 | 50 |
| Traffic lights on | 11 (15%) | 9 (8%) |
| No traffic lights | 60 | 104 |

**Supplementary Figure 1.** Risk of road traffic accident leading to injuries (A) in cancer and control cohorts. Accidents without injury are shown in (B), with data starting from 2017.
